# Using Total Worker Health^®^ Implementation Guidelines to Design an Organizational Intervention for Low-Wage Food Service Workers: The Workplace Organizational Health Study

**DOI:** 10.3390/ijerph18179383

**Published:** 2021-09-06

**Authors:** Eve M. Nagler, Elisabeth A. Stelson, Melissa Karapanos, Lisa Burke, Lorraine M. Wallace, Susan E. Peters, Karina Nielsen, Glorian Sorensen

**Affiliations:** 1 Center for Community-Based Research, Dana-Farber Cancer Institute, Boston, MA 02215, USA; estelson@g.harvard.edu (E.A.S.); melissa.pember@gmail.com (M.K.); Lisa_Burke@dfci.harvard.edu (L.B.); Lorraine_Wallace@dfci.harvard.edu (L.M.W.); sepeters@hsph.harvard.edu (S.E.P.); Glorian_Sorensen@dfci.harvard.edu (G.S.); 2 Department of Social and Behavioral Sciences, Harvard T.H. Chan School of Public Health, Boston, MA 02115, USA; 3 Institute of Work Psychology, Sheffield University Management School, University of Sheffield, Sheffield S10 1FL, UK; k.m.nielsen@sheffield.ac.uk

**Keywords:** total worker health, intervention development, working conditions, occupational safety and health, food service workers, wellbeing, pain and injury, work environment

## Abstract

Total Worker Health^®^ (TWH) interventions that utilize integrated approaches to advance worker safety, health, and well-being can be challenging to design and implement in practice. This may be especially true for the food service industry, characterized by high levels of injury and turnover. This paper illustrates how we used TWH Implementation Guidelines to develop and implement an organizational intervention to improve pain, injury, and well-being among low-wage food service workers. We used the Guidelines to develop the intervention in two main ways: first, we used the six key characteristics of an integrated approach (leadership commitment; participation; positive working conditions; collaborative strategies; adherence; data-driven change) to create the foundation of the intervention; second, we used the four stages to guide integrated intervention planning. For each stage (engaging collaborators; planning; implementing; evaluating for improvement), the Guidelines provided a flexible and iterative process to plan the intervention to improve safety and ergonomics, work intensity, and job enrichment. This paper provides a real-world example of how the Guidelines can be used to develop a complex TWH intervention for food service workers that is responsive to organizational context and addresses targeted working conditions. Application of the Guidelines is likely transferable to other industries.

## 1. Introduction

Ensuring worker safety, health, and well-being is critical for employees and employers, since this can directly and indirectly contribute to enterprise outcomes, such as productivity and turnover [1]. Given the diversity of worksites, work arrangements, and challenges to worker well-being, the National Institute for Occupational Safety and Health developed Total Worker Health^®^ (TWH) as a holistic approach to promoting and protecting worker safety, health, and well-being. TWH is defined as “policies, programs, and practices that integrate protection from work-related safety and health hazards with promotion of injury and illness-prevention efforts to advance worker well-being” [2]. Central to TWH is the idea that integrated interventions and approaches that address policies, programs, and practices within the work environment are critical to modifying the multifaceted and interwoven factors that affect worker safety, health, and well-being [1,3]. These approaches are also important for identifying and improving working conditions and changes in work arrangements shaped by the future of work [4].

However, designing these approaches can be difficult in practice, especially since some employers perceive psychosocial working conditions to be more challenging to manage than physical health and safety risks [5,6,7,8]. To support the dissemination and utilization of TWH approaches, the Center for Work, Health, and Well-being at the Harvard T.H. Chan School of Public Health (“the Center”) developed a set of TWH guidelines titled *Implementing an Integrated Approach: Weaving Worker Health, Safety, and Well-being into the Fabric of Your Organization* (“the Implementation Guidelines”) [9]. The Implementation Guidelines were developed to help workplaces plan, implement, and evaluate integrated approaches that address working conditions to improve worker safety, health, and well-being. While the Implementation Guidelines can be utilized by individuals in diverse industry sectors, application of the Implementation Guidelines requires translation to specific organizational goals and contexts, as well as workplace needs and requirements. Our goal is to help practitioners and researchers successfully design and implement TWH interventions for their organizational contexts and workplace settings. To this end, this paper illustrates how we applied specific elements of the Implementation Guidelines to develop and implement an organizational TWH intervention to improve the safety and well-being of low-wage food service workers, a workforce that experiences high rates of injury and turnover [10]. Using this industry example, this paper aims to contribute to the body of TWH research-to-practice literature of how to develop interventions that address working conditions; it also provides transparency around the decision-making processes and methods used to guide the stages of intervention development, as called for by other researchers [11,12].

### 1.1. The Implementation Guidelines

The Implementation Guidelines are based on a rich foundation of research, practice, and a conceptual model developed earlier by our research team [1]. The model is grounded on several interdisciplinary theoretical perspectives, including the social ecological model [13,14], social contextual model of health behavior change [15,16], hierarchy of controls [17,18,19], and participatory frameworks [20]. At the core of the Implementation Guidelines is the use of policies and practices to improve working conditions to address underlying systemic issues that are often the root causes of worker health, safety, and well-being concerns [9]. Working conditions can include factors in the physical environment, such as the equipment used or how the built environment is laid out; job demands such as the pace and amount of work, which may be influenced by staffing levels; and psychosocial factors such as supervisor support, teamwork, and job stress [9].

The Implementation Guidelines outline four stages of an iterative and integrated process to (1) foster buy-in and collaboration among worksite stakeholders, and (2) plan, (3) implement, and (4) evaluate a TWH approach. At the center of this process are the six key characteristics of an integrated approach that are important to the success of a TWH initiative and guide decision-making at each stage: (1) leadership commitment; (2) participation; (3) policies, programs, and practices focused on positive working conditions; (4) comprehensive and collaborative strategies; (5) adherence; and (6) data-driven change [1,9]. We recently applied the Implementation Guidelines to develop and test an integrated, organizational intervention to improve worker safety, health, and well-being in a high-risk sector (low-wage food service) [21], a particularly challenging undertaking due to the hierarchical organizational structure and economic and social vulnerability of frontline staff in this setting [22,23,24,25,26].

### 1.2. Low-Wage Food Service Workers and the Workplace Organizational Health Study

Compared to other industries, work-related injury and illness among workers in the food service industry is among the highest in the United States [10,27,28]. Among the 12 million workers employed in the food service industry in 2019, the incidence of occupational injuries requiring days missed from work was 1.9 per 100 workers employed in special food services such as contract work [10]. In comparison, workers employed in manufacturing and construction industries—industries known for their high rates of injury—had incidences of 2.0 and 1.7 per 100 workers, respectively, while business and professional service sectors reported 0.7 injuries per 100 workers [10]. 

The work environments found in the food service industry can affect the safety, health, and well-being of frontline staff. For example, employees may experience strains and sprains because of repetitive movements (e.g., chopping and mixing), pushing carts, or prolonged standing, such as for cashiers. Similarly, jobs can involve awkward postures, bending, and lifting heavy equipment or items. Workers can slip, trip, and fall because of slick floors and clutter. They also suffer cuts from sharp objects and burns from exposure to hot stoves, ovens, cooking oils, and steam [27,28,29,30,31]. In parallel, employees also face organizational conditions and high psychosocial demands from responding to a fast-paced work environment, time pressures, and limited rest breaks from producing meals (e.g., breakfast and lunch) on a tight schedule and responding to catering requests [27,30,31,32,33,34]. These conditions may contribute to the fact that job separation is 75% higher among food service workers compared to the 2019 cross-industry average [35].

While many of these worker safety, health, and well-being outcomes are rooted in the working conditions of the food service work environment, warranting interventions that focus on system-wide, organizational improvements, few have been tested and are available in the existing literature [36,37,38,39,40]. This need prompted a large, multinational company (“Company”) that provides food services through contractual arrangements to approach the Center to develop and test approaches to improve the health, safety, and well-being of their frontline workers. This partnership resulted in the development of a TWH organizational intervention focused on improving working conditions related to decreasing pain and injury and increasing worker well-being. Because of the Implementation Guidelines’ sharp focus on using policies and practices to improve working conditions, we used them as a framework to develop the intervention. The purpose of this paper is to illustrate the process of using the Implementation Guidelines to develop and implement a TWH intervention as part of The Workplace Organizational Health Study [21]. In doing so, we additionally aim to provide an approach to develop TWH interventions that can be used by practitioners and researchers in other industry sectors.

## 2. Materials and Methods

### 2.1. Study Setting and Population 

The intervention was designed for five of the Company’s food service worksites, located in corporate settings in the Greater Boston area. Each site had 7–22 frontline workers and a site manager who was responsible for operations and supervision of frontline workers. The Company’s project champion, who was a member of the national leadership team, contacted the Center about initiating the study and provided corporate-level support throughout the project. Company leadership at the district and national levels also participated in intervention development at multiple stages in the process. 

### 2.2. Design Team

To develop the intervention utilizing the Implementation Guidelines, we established a multi-disciplinary research team, comprised of Center social and behavioral scientists, occupational safety and health experts, and public health practitioners. To inform the intervention development process, we conducted formative research in five of the Company’s worksites that were not part of the planned intervention study [41]. The research team met weekly and collaborated regularly with the company national project champion, which provided an opportunity for feedback and discussion reflecting diverse and extensive intervention expertise. Throughout intervention development, the team met with Company leaders at different levels to incorporate their expertise into the design of the intervention, learn about existing Company resources, and garner buy-in. We used an iterative process in which we applied what we learned at each stage of intervention development to inform the next, a process we have used to successfully develop other interventions [42,43]. This allowed us to modify the intervention as needed based on experience and feedback and to ensure stronger organizational fit. 

We also kept comprehensive intervention development and meeting notes, which allowed us to document key decisions and any adaptations made to the intervention as it was implemented. Intervention staff documented barriers and facilitators encountered after each intervention contact. These notes were securely stored in a shared folder, accessible to the team, allowing for review of decision points and easy access to information to share with the larger investigator team and project champion.

### 2.3. Using the Implementation Guidelines to Develop the Intervention

We used the Implementation Guidelines to develop the intervention in two main ways: first, we used the six key characteristics of an integrated approach to create the foundation of the intervention [44]; second, we used the four stages to guide iterative and integrated intervention planning.

#### 2.3.1. Key Characteristics

The six key characteristics described in the Implementation Guidelines are indicators of best practices for protecting and promoting worker safety, health, and well-being, as defined in Table 1 [9]. We used the key characteristics to guide decision-making related to: (a) who to involve? Leadership commitment (key characteristic 1) and broad participation of stakeholders, especially workers, (key characteristic 2) are pivotal to intervention success; (b) what to change? Using policies and practices to improve working conditions (key characteristic 3) is fundamental to the TWH approach and formed the basis of our initiative; (c) how to design the intervention? Best practices, such as implementing comprehensive and collaborative strategies (key characteristic 4) to foster employee engagement; promoting adherence to federal and state regulations and ethical norms (key characteristic 5); employing data-driven change to guide decision-making and improve an initiative (key characteristic 6) were all considered in the design of the intervention to change working conditions.

#### 2.3.2. Stages

The Implementation Guidelines, organized into four stages of an integrated approach, provide processes to plan an intervention and strategies to change working conditions (Table 2). For example, the Implementation Guidelines recommend having employee-manager teams work collaboratively to identify relevant working conditions and develop action plans. 

The application of the Implementation Guidelines to intervention planning is described in the Section 3. 

**Figure 1:**
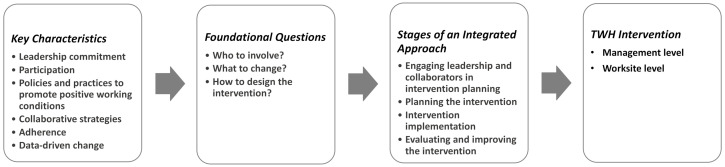
The Implementation Guideline’s six key characteristics helped us answer the three questions that created the foundation of intervention. Those three questions informed our decision-making at each of the four stages from the Guidelines, leading to the design of a multilevel intervention.

Figure 1 illustrates the overall process used: the key characteristics helped us answer the three questions that created the foundation of the intervention; the questions guided decision-making at each of the four stages, leading to the design of the organizational intervention.

## 3. Results

### 3.1. Applying the Key Characteristics

Who to involve? Using knowledge of the organizational setting and study outcomes determined based on our formative research [41], we collaborated with the Company to determine who to involve in the intervention. Specifically, we selected (1) site-level managers, since middle-managers are crucial to intervention success; (2) site-level frontline workers (chefs, cooks, food preparers, servers, dishwashers, and cashiers), given the importance of employee participation in all phases of planning and implementing an intervention; (3) leadership, as different types and levels of leadership were needed to develop, implement, and support this intervention. For example, we collaborated with representatives of safety and health to ensure that our intervention aligned with approaches already being used within the Company. Similarly, we engaged human resources and operations leadership in order to build upon strategies already in place. Collaborating with the Company’s project champion throughout the project was also crucial to maintain corporate support. 

What to change? Based on a literature review of organizational interventions [45], formative research, including interviews with managers, focus groups with frontline workers, and a collaborative participatory process with the Company [41], we targeted three working conditions: (1) safety and ergonomics (equipment use, slips and falls, prolonged standing, and lifting and carrying demands); (2) work intensity (the pace of work and demands placed on employees); and (3) job enrichment (providing on-going job performance feedback and coaching and identifying opportunities for career advancement). Additionally, we identified four essential intervention elements (leadership commitment, participation of stakeholders at all levels of the Company, communication, and tailoring for fit), defined as mechanisms for achieving the intervention’s intended effects that are well-aligned with our six key characteristics [41].

How to design the intervention? Given the targeted working conditions, essential elements, and knowledge of the context, we selected collaborative strategies (key characteristic 4) to enhance communication between employee levels, such as by promoting manager/worker action planning. We also decided to focus on data-driven change (key characteristic 6) to inform how we would tailor the intervention to “fit” the Company’s organizational context. Because the Company was already adherent to federal and state regulations (key characteristic 5), we decided to build on the Company’s strong safety program as we developed the intervention.

### 3.2. Applying the Stages 

The following summary of our experience developing the intervention is organized based on the four stages outlined in the Implementation Guidelines. For each stage, we describe the Implementation Guidelines’ recommendations, how these were applied to the development of our intervention, and considerations for others who may use the Implementation Guidelines to plan an intervention. 

#### 3.2.1. Stage 1: Engaging Leadership and Collaborators in Intervention Planning

- What the Implementation Guidelines Recommend.

Having broad-based support—from top leadership and middle management to workers at all levels—is crucial to implementing an organizational intervention to create safer and healthier workplaces [9,46,47]. The Implementation Guidelines recommend working with multiple groups across sectors to achieve ongoing support. Leadership buy-in sets the stage for an intervention by engaging management at different hierarchical levels of the company, committing resources, and creating a supportive environment. Middle managers directly manage both the day-to-day workflow to ensure the company’s work gets completed, and supervise employees, which entails creating working conditions and a work environment in which everyone can succeed. Soliciting and acting upon employees’ input about working conditions through regular and continuous communication can improve the fit of strategies selected to address them and foster future participation. Employees may also feel more engaged, which contributes to their health, safety, and well-being [48]. Managers can also promote engagement by frequently communicating with employees, obtaining ongoing feedback, and “recognizing the value of [employees’] work” [9].

b. How we applied the Implementation Guidelines to engaging leadership and promoting collaboration.

From our literature review, interviews, focus groups, and collaboration with the Company, we learned several insights that influenced how we engaged leadership and promoted broad-based collaboration in the intervention [41]. First, we learned that national leadership wanted more frequent communication between site managers and their frontline workers, above and beyond annual performance reviews. Specifically, they wanted a participatory process or tool that could be scaled across the organization. 

Second, we learned that each level of Company management had different decision-making authority for how policies and practices were implemented at the national, district, and site levels. We also learned that the work environment and food service operations (e.g., size of the kitchen and number of catering requests) varied greatly from site to site within the Company. This informed our decision to work with each site to assess working conditions and plan for site-specific changes, given their unique contexts. In parallel, we recognized that intervention was needed at the leadership level to leverage the different management levels’ abilities to change policies and practices to support site changes.

Third, we learned that different leadership groups (e.g., safety, human resources, and operations) had distinct priorities that we would need to build upon throughout intervention planning. Specifically, we decided to engage these groups in relevant parts of intervention development (e.g., safety and ergonomics) to learn how Company policies, practices, and resources could be leveraged, and to solicit their ideas for changing working conditions. 

Given the key role of middle managers to intervention success, we also decided early on to work closely with the site managers to better understand working conditions from their perspective, tailoring the intervention to fit their context, and engaging them in participatory intervention delivery. We created tools, such as action planning forms, that were sensitive to their time and easy to complete. 

To enhance collaboration and communication between site managers and frontline workers, we asked managers to work with their employees to assess working conditions and develop and implement plans to address these conditions. To facilitate this process, we developed a tip sheet for managers on how to engage workers at different stages. We also added discussion questions for managers to ask frontline workers during “huddles” (short team meetings) to continually engage them in the intervention.

c. Considerations for intervention developers.

It is important to take the time to understand who has decision-making authority that will influence the targeted changes and to align with their priorities early on. This will help ensure developers are engaging the appropriate individuals who can support the intervention process and changes in working conditions. Identifying and leveraging existing systems, processes, and resources can help embed the intervention into the way an organization does business. Additionally, it is important to obtain employee feedback in a way that makes the people involved in the intervention feel comfortable, such as soliciting anonymous comments or holding meetings with different stakeholder groups to allow people to speak candidly.

#### 3.2.2. Stage 2: Planning the Intervention

- What the Implementation Guidelines recommend.

Well-crafted plans are the backbone of successful workplace initiatives [4]. At this stage, the Implementation Guidelines recommend gathering information about working conditions, prioritizing actions, developing an action plan to address objectives, and evaluating and monitoring the plan [9]. This process can be an effective platform for implementing organizational interventions [49,50] by identifying “what works” in a company to align intervention approaches with organizational priorities and promote positive changes that build upon existing practices.

b. How we applied the Implementation Guidelines to intervention planning.

Using information learned in Stage 1, we planned the intervention with the following components. First, each working condition (safety and ergonomics, work intensity, and job enrichment) would be addressed in its own module, to be delivered sequentially. Because the three working conditions were distinctly different, each required its own assessment and planned actions as recommended in the Implementation Guidelines. While all sites would address the same working conditions, the solutions implemented for each working condition would be site-specific. With frontline worker input, each site manager would assess needs and assets related to each working condition. A research team member would then interview the site manager and generate a report with recommendations to assist priority-setting. The safety and ergonomics assessment also included a walkthrough conducted by an industrial hygienist to identify site-specific areas of improvement.

Second, each site would develop and implement action plans based on their site-specific solutions, using the assessment report, planning tools, and consultation provided by a member of the research team. However, site managers would also be encouraged to share and learn from each other. For example, for the Work Intensity module, we convened site managers on a call to share experiences related to the pace of work and the demands placed on both managers and employees, discuss strategies to address challenges, and identify common themes that could be shared with district managers and senior leadership. 

Third, training would be conducted to enhance employee engagement. For example, for the Job Enrichment module, we trained site managers on ways to provide coaching and feedback, and how to work with employees to improve performance and achieve career growth. Finally, leadership would be encouraged to support sites with system-level changes. National leadership and district-level managers would review aggregate findings from the site assessments for each working condition and determine what cross-site policies and practices could be improved to support site plans. They would also identify company resources to use during the intervention and address any challenges to intervention implementation. To build leadership buy-in and commitment, we engaged leaders in the planning and development of the intervention using the following strategies. 

Building on existing practices and “what works”: We worked with leadership to identify and tailor an existing company action planning form for sites to use during the intervention. The form had fields to record areas to improve, SMART (Specific, Measurable, Attainable, Relevant, and Time-bound) objectives, tactics, persons responsible, due dates, resources required, suggested policies to implement and plans for evaluation, and how to sustain each area of improvement. We utilized scripts for “huddles” between site managers and frontline workers (a commonly used Company communication tool used at sites), to support the site manager’s leadership and communications with her/his team during the intervention. For the Job Enrichment module, we modified an existing tool—“2 + 2 Coaching and Feedback”—for site managers to use with frontline workers. Previously, this communication tool had been used for the site manager level and above. 

Building on organizational priorities: Because leadership expressed pride in their Company’s strong safety program, we started the intervention with the safety and ergonomics module to help build trust and establish early successes. This also reinforced the TWH approach to first prioritize a “hazard-free work environment” [2]. Knowing Company leadership wanted to enhance employee engagement, we sought ways to foster participation and communication between site managers and employees during planning. Specifically, we sought to make both the managers and employees “agents of change” by collaborating to identify how working conditions impacted their safety and well-being, brainstorm solutions, and develop and implement action plans to change working conditions that were tailored to fit their sites’ assets and experiences. 

**Figure 2:**
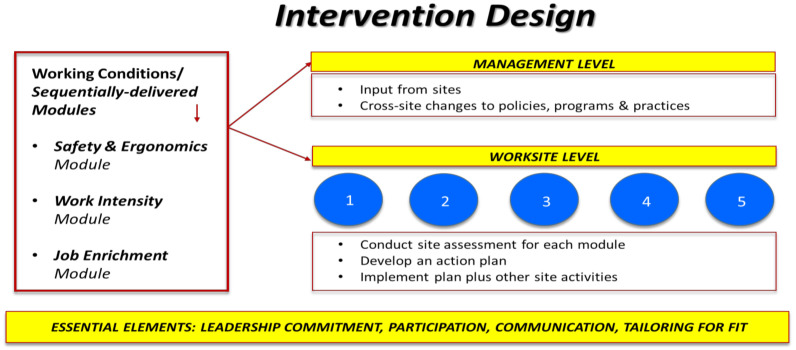
Organizational intervention for improving safety, health, and well-being of low-wage food service workers [26].

Incorporating each of these components, we created a 13-month intervention with three modules, focused on two levels (management and site level), and with implementation of each module lasting approximately four months (Figure 2). Protocols, including objectives and supporting materials, were developed for each module, and delivered onsite by a research team member assigned to be the primary representative of the project within each worksite. Ongoing meetings and collaboration with district and national leaders occurred across the 13-month study period.

c. Considerations for intervention developers.

Intervention developers can build on existing practices and procedures to help make a “new” initiative feel familiar and embed the activities into the way a company does business. Focusing on organizational priorities during the planning stage, and engaging relevant policy makers in the process, can also help generate leadership support. Intervention developers might also consider conducting assessments to capture both strengths and challenges and help workplaces develop solutions that build upon existing organizational resources.

#### 3.2.3. Stage 3: Planning for Implementation of the Intervention

- What the Implementation Guidelines recommend.

Once an intervention is developed, the Implementation Guidelines offer suggestions to facilitate implementation, including starting small and communicating about intervention progress. Implementing one or a few simple tactics at a time allows worksites to build on incremental improvements, demonstrate progress, and learn from early wins, which can generate interest and potential sustainability [51]. Ongoing communication between organizational leadership, middle management, and workers helps maintain interest and support for the approach. For example, listening to and acting on employee input can help workers feel involved and part of the process, which can foster participation. Keeping leadership abreast of how the intervention is unfolding can also identify new solutions during implementation.

b. How we applied the Implementation Guidelines to planning intervention implementation.

To implement the intervention, the research team member would first visit the site to introduce the module and review the associated intervention materials with the site manager. They would also brainstorm with the site manager about developing and implementing site-specific action plans based on information gathered during the site assessment. The research team member would provide tools for developing the action plan and engage workers in the process, offer guidance to prioritize actions, and provide ongoing consultation and technical support to site managers during in-person and phone meetings. As an implementation strategy, this consultative approach has been shown to be critical to the uptake and quality of intervention implementation [52,53]. As recommended by the Implementation Guidelines, the research team member would encourage site managers to pick something from their action plan that they could feasibly implement at low or no cost. This incremental strategy would allow sites to experience early successes and build momentum as they conduct assessments and develop action plans for subsequent modules. 

At the leadership level, we designed the intervention to support ongoing communication about intervention progress though meetings with senior leadership and district managers. These meetings would also help identify existing resources and ensure the intervention aligned with the priorities of the different leadership groups. Summary reports of aggregate findings from the site assessments would facilitate leadership’s implementation of policies and practices to address common challenges experienced by sites for each working condition. 

To support leadership’s indicated priority of enhancing employee engagement, we decided to conduct a training for all site managers during the Job Enrichment module. The training focused on how to provide coaching and feedback and work with employees to improve performance and identify opportunities for career growth. In discussions with Human Resources, we learned the Company had an existing 2 + 2 Coaching and Feedback tool that was already used to provide feedback to management-level employees, but there was no equivalent tool to provide feedback to frontline employees. The 2 + 2 model is based on a 15 min conversation, during which the manager and employee discuss two things the employee is doing well and two things the employee could be doing more or less of to enhance or improve performance or career growth. The conversation concludes with the manager and employee agreeing to specific actions and next steps, including a plan for follow-up. These 2 + 2 conversations had been effective at the management level of the Company, because they are short, simple, and scripted. This approach supported collaboration, communication, and participation by helping managers (a) incorporate concrete feedback into their coaching and (b) collaboratively work with their employees to develop next steps. 

We decided to tailor the 2 + 2 Coaching and Feedback tool for use with frontline workers and created a 45 min, interactive webinar training for site managers, with Company leadership setting the expectation that managers would use this tool with their employees during their annual performance reviews and regularly during the year. Accordingly, this adapted Company tool promoted each of the four essential elements of our intervention: (1) leadership commitment: Human Resources leadership communicated their support and use of the modified 2 + 2 model with frontline employees, based on its previous use with managers. (2) participation: 2 + 2 Coaching and Feedback encouraged improved two-way discussions and invited employee participation and input. (3) communication: 2 + 2 Coaching and Feedback encouraged short, more frequent conversations between site managers and workers, which aligned with senior management’s goal of enhanced communication. (4) tailoring for fit: 2 + 2 Coaching and Feedback had previously been used in other parts of the Company and at different levels, so it was already aligned with the organizational culture.

c. Considerations for intervention developers.

Providing hands-on and ongoing consultation can facilitate intervention implementation by helping organizations develop action plans, ensure organizational and site-specific priorities are clear, and decide which actions to initiate first. Intervention developers should also consider using multiple intervention strategies and communication modalities (e.g., in person visits, phone calls) to accommodate the pace of work and schedules of people implementing an intervention. Others may also find it beneficial to start small to build early success, and to identify existing resources that provide established platforms to further the intervention goals.

#### 3.2.4. Stage 4: Planning for Evaluating and Improving the Intervention

- What the Implementation Guidelines recommend.

Evaluation and continual improvement are fundamental to the successful implementation of integrated, organizational approaches and can have implications for long-term success [54]. The Implementation Guidelines recommend using both quantitative (e.g., surveys) and qualitative (e.g., focus groups, interviews, or informal conversations with workers) data to understand what took place and why from the perspective of employees at all levels of an organization. Ongoing evaluation during implementation is key to provide data for making mid-course corrections, which can influence the intervention’s ultimate success in improving outcomes [55,56]. Because implementation of an intervention may not go exactly as planned, the Implementation Guidelines help intervention developers identify where changes are needed and focus on incremental improvements. Additionally, monitoring the implementation process helps intervention developers learn not only what strategies are working, but also why, which gives essential insight to tailor the intervention to fit the context.

b. How we applied the Implementation Guidelines to plan for intervention improvement.

We planned for ongoing monitoring and using data to continually improve the intervention fit with the organizational context. At the site level, each module would begin with an assessment of a specific working condition. Site managers would be encouraged to use these data to tailor solutions to fit their site. To enhance employee participation, we encouraged discussions and devised alternative ways for employees to provide confidential feedback, such as using suggestion boxes. At the leadership level, we planned to keep district managers and senior leaders abreast of how the intervention was unfolding, the successes achieved, and any challenges to implementation. We also anticipated making data-driven modifications to the overall intervention as needed. To achieve this, we created a tracking system of the intervention process and to what degree the intervention was actually implemented [26] to help us develop a deeper understanding of intervention implementation at both the site and leadership levels. This system included observations and reflections from the research staff members who served as the primary contacts for the intervention at the five sites. Finally, through ongoing collaboration and listening to stakeholders at all levels, we would be able to learn what worked, what did not work, and why. As recommended by the Implementation Guidelines, collection of real-time data would facilitate continuous improvement of the intervention.

c. Considerations for intervention developers.

It is important to remember that intervention development and implementation is an iterative process. Interventions are frequently not implemented as planned, requiring flexibility to change and adapt the intervention as needed to accommodate the organizational or worksite context [57]. To achieve this, it is helpful to build opportunities for reflection on potential adaptations as part of the intervention plan. Building observations and dialogues into intervention monitoring can help intervention developers understand which intervention activities are going well and which ones are not. Data collection can also function as a useful communication tool so workers know what is happening, and leaders learn about successes and can identify where more resources could be helpful.

## 4. Discussion

Participatory TWH interventions can be challenging to design and implement in real-world settings, especially in low-wage, fast-paced, high-attrition industries such as food service [35]. While an integrated approach to improving the programs, policies, and practices which structure worker health outcomes is imperative, there are few examples accessible to researchers and practitioners, such as human resources personnel, occupational health professionals, and union representatives, to successfully plan and implement TWH interventions. The goal of this paper is to provide a pragmatic step-by-step example of how to use the Harvard T.H. Chan School of Public Health Center for Work, Health, and Well-being’s TWH Implementation Guidelines to develop a complex TWH organizational intervention.

Using the Workplace Organizational Health Study as an illustrative example, the Implementation Guidelines helped us to design the intervention in two central ways. First, the six key characteristics of an integrated approach guided decisions that laid the foundation for the intervention, including (a) who to involve, guided by the characteristics of leadership commitment and participation of stakeholders across the Company, especially frontline workers; (b) what to change, by identifying working conditions and related Company policies and practices through preliminary research and collaboration with the Company; (c) how to design the intervention to include collaboration across Company levels and ongoing monitoring to promote data-driven modifications to the intervention as needed. Second, the Implementation Guidelines—organized into four stages of an integrated approach—provided a process to plan the intervention using strategies to change working conditions. The planning process culminated in a 13-month intervention implemented at both leadership and site levels to change our three working conditions (safety and ergonomics, work intensity, and job enrichment), as described in detail elsewhere [26]. 

The Implementation Guidelines were designed for organizations to create TWH approaches. This research supports and advances the fundamentals of TWH in several important ways. First, it harmonizes with the TWH definition and key tenant of a TWH approach: “focusing on addressing system-level or environmental determinants of health before individual-level approaches” [58]. Second, it strengthens the five Defining Elements of TWH: (1) demonstrate leadership commitment to worker safety and well-being—we prioritized leadership engagement throughout all modules; (2) design work to reduce safety hazards—as in the safety and ergonomics module; (3) promote worker engagement—through assessments of working conditions, action planning, and training site managers to have ongoing conversations with their employees related to performance and career growth; (4) ensure confidentiality and privacy of workers—by using suggestion boxes for workers to provide confidential feedback and providing aggregate site assessments to leadership; (5) integrating relevant systems—by leveraging Company systems and resources, such as modifying existing planning forms and adapting the 2 + 2 Coaching and Feedback tool for use with frontline workers [59]. Finally, our selection of working conditions to address in the intervention supports issues NIOSH has identified as relevant to advancing worker well-being through TWH. Specifically, our approach to safety and ergonomics supports TWH issues related to “control of hazards and exposures,” the work intensity focus aligns with “organization of work” issues, and the inclusion of job enrichment supports the issue of “career and skills development” [59]. 

In a field, such as food service, where there are few examples of successful participatory TWH organizational interventions, providing transparency around our process for developing a complex intervention at multiple levels and with different stakeholders provides a systematic process and structure that practitioners and researchers can use. In recent years, there has also been a call to publish intervention development studies—particularly complex interventions such as this one—and for transparency in reporting the decision-making processes, experiences, and methods used in the stages of intervention development [11,12]. This sharing can foster cross-disciplinary learning and help intervention developers replicate the processes and avoid recurring pitfalls [12]. We believe this paper makes an important contribution to this effort.

Related to the future of work, designing integrated approaches is especially important considering evolving changes to the work environment, employment relationships, and how work is performed [4,60,61]. The COVID-19 pandemic accelerated some future of work changes. The Implementation Guidelines can help organizations plan integrated approaches that evaluate and remedy emerging changes in working conditions that impact worker safety, health, and well-being, allowing them to be more agile and resilient in times of change, such as the pandemic. For example, communication mechanisms became very important so that new guidelines and changes to work could be communicated rapidly to workers; supervisors were essential to helping workers navigate these changes. Additionally, implementing remote work policies impacted onsite contractors, such as food service workers, resulting in decreased employment stability and job security for these workers. 

The use of the Guidelines to inform intervention development, content and strategies is supported by other work conducted by the Center in different sectors. For instance, we recently used the Implementation Guidelines’ six key characteristics to organize TWH recommendations for worksites on practical ways they could address worker safety, health, and well-being during the COVID-19 pandemic [62]. The Center’s Implementation Guidelines were also used in conjunction with other theoretical models to design a participatory organizational intervention for subcontractors in the commercial construction industry [43]. Additionally, our decision to provide consultation and technical assistance to sites is supported by a recent study in which we tested the practical utility of a TWH capacity-building suite of services, including the Implementation Guidelines, to help worksites develop TWH action plans [63]. Outside of TWH, we have employed this strategy to help program implementers feel comfortable and confident in their roles [42,64]. 

### 4.1. Strengths and Limitations

This study has several strengths. To our knowledge, this is the first application of the Implementation Guidelines to create a TWH intervention for low-wage food service workers, focused on improving the conditions of work. It provides practitioners and researchers with a concrete example of how to develop a TWH organizational intervention in partnership with a multinational corporation. We kept comprehensive intervention development and meeting notes, which documented decisions and adaptations made to inform future interventions. This was not only helpful when iteratively developing the intervention during this study, but also to reflect on our process and to develop considerations for others who may use the Implementation Guidelines; these considerations are provided in this paper. We encourage other intervention developers to keep similar notes. Notably, we used the Implementation Guidelines as part of an academic research study. Given that the Implementation Guidelines were developed for use by workplaces, our experience speaks to their broad applicability in both research and practice settings. 

Despite these strengths, there are limitations to this study. We designed the intervention for sites to assess working conditions, develop action plans, and implement changes for three working conditions over the course of 13 months. However, another recent study reported that it may take worksites approximately nine months to form an integrated team and craft a TWH action plan with on-going technical assistance [63], suggesting we may have been too ambitious in our intervention timeline [26]. We also realize the intervention development process, design, and choice of key characteristics may look different for organizations that are creating an intervention for one site verses tailoring the intervention for multiple sites. Since this was a research study, we selected, in advance, the working conditions that the sites would address. In practice, worksites might identify different sets of working conditions to target during the planning stage of the intervention. Application of the Implementation Guidelines may also look different when applied from within the organization rather than by researchers in collaboration with organization representatives.

### 4.2. Future Directions

While we have found the Implementation Guidelines to be transferable across industries, future efforts can be made to document how the Implementation Guidelines are used in other real-world settings and work contexts. For example, future research might compare the implications of these Implementation Guidelines for employers across different sectors, varying by size and with different readiness for change. Approaches to using a participatory process may also vary depending upon existing resources, leadership commitment, and levels of employee engagement. We believe the provision of concrete examples that transparently document the intervention development processes and decision-making, and provide considerations for future intervention developers can be a useful model to bridge research and practice gaps. 

## 5. Conclusions

This translational research-to-practice paper demonstrates how we used the Harvard T.H. Chan School of Public Health Center for Work, Health, and Well-being’s Implementation Guidelines to develop an organizational TWH intervention to improve the safety and well-being of low-wage food service workers. We used the Implementation Guidelines’ key characteristics to establish who to involve, what to change, and how to design the intervention throughout a four-stage process. For each stage, the Implementation Guidelines provided suggestions and strategies to develop an evidence-informed intervention. The Implementation Guidelines can be used by both practitioners and researchers to develop interventions that are responsive to the organizational context and address targeted working conditions to improve worker safety, health, and well-being. Efforts such as this to translate theory-based Implementation Guidelines into pragmatic processes and considerations for both practitioners and researchers is an important step forward to develop TWH interventions across industry sectors. 

## Figures and Tables

**Table 1:** Six key characteristics of a TWH integrated approach [9].

Key Characteristic	Definition from the Implementation Guidelines
1. Leadership commitment	Leadership makes worker safety, health, and well-being a clear priority for the entire organization. They drive accountability and provide the necessary resources and environment to create positive working conditions.
2. Participation	Stakeholders at every level of an organization, including organized labor when applicable, help plan and carry out efforts to protect and promote worker safety, health, and well-being.
3. Policies, programs, and practices focused on positive working conditions	The organization enhances worker safety, health, and well-being with policies, programs and practices that improve working conditions.
4. Comprehensive and collaborative strategies	Employees from across the organization work together to develop comprehensive safety, health, and well-being initiatives.
5. Adherence	The organization adheres to federal and state regulations, as well as ethical norms, that advance worker safety, health, and well-being.
6. Data-driven change	Regular evaluation guides an organization’s priority setting, decision making, and continuous improvement of worker safety, health, and well-being initiatives.

© 2017 Harvard T.H. Chan School of Public Health Center for Work, Health, and Well-being.

**Table 2:** Four stages of an integrated TWH approach [9].

Stage	Description from the Implementation Guidelines
1. Engaging Leadership and Collaborators	Buy-in and collaboration from across the organization are important. Seek top leadership support early on, encourage collaboration, work closely with middle managers, and give workers clear opportunities to participate.
2. Planning	Successful initiatives start with a clear plan. Define the goal and choose SMART (Specific-Measurable-Achievable-Relevant-Time Bound) objectives. Define working conditions; gather and analyze worksite information; select tactics; create an action plan; identify required resources.
3. Implementation	Changes to policies, practices, and programs play out in the workplace. To facilitate implementation, start small, communicate about progress, and conduct training.
4. Evaluation and Improvement	Monitor and analyze data to measure success and improve an initiative. Use a variety of data collection methods, evaluate as needed, and communicate findings frequently.

© 2017 Harvard T.H. Chan School of Public Health Center for Work, Health, and Well-being.

## Data Availability

The data presented in this study are available on request from the corresponding author. The intervention development and meeting notes data are not publicly available due to confidentiality concerns of the collaborating Company.
